# Demographic bias in public remote photoplethysmography datasets

**DOI:** 10.1038/s41746-025-01973-9

**Published:** 2025-10-02

**Authors:** Maksym Bondarenko, Carlo Menon, Mohamed Elgendi

**Affiliations:** 1 Biomedical and Mobile Health Technology Research Lab, ETH Zürich, Zürich, Switzerland; 2 School of Computation, Information and Technology (CIT), Department of Computer Science, TUM, Munich, Germany; 3 Department of Biomedical Engineering and Biotechnology, Khalifa University of Science and Technology, Abu Dhabi, United Arab Emirates; 4 Center for Biotechnology, Khalifa University of Science and Technology, Abu Dhabi, United Arab Emirates

**Keywords:** Biomarkers, Medical research, Risk factors

## Abstract

Remote photoplethysmography (rPPG) is gaining traction for non-contact heart rate estimation, yet most publicly available datasets are demographically biased. In this study, we analyze 100 rPPG studies, providing the first quantitative cross-model audit of demographic bias in rPPG and demonstrating significant underrepresentation of darker skin tones and gender imbalance. Our findings reveal how this bias limits model fairness and accuracy and propose steps to improve dataset inclusivity and algorithmic robustness.

## Demographic biases in public rPPG datasets

Machine-learning–driven remote photoplethysmography (rPPG) has revolutionized heart-rate (HR) estimation by enabling non-contact monitoring with standard RGB cameras^1^. Huang et al. (2023) categorize a wide range of visual contactless physiological monitoring (VCPM) scenarios—newborn and ICU monitoring, telemedicine, elderly and home care, fitness and rehabilitation, face anti-spoofing, and pilot/astronaut health assessment^2^. Di Lernia et al. (2024) demonstrate that rPPG can accurately recover HR even in uncontrolled, “in the wild” online settings^3^.

Despite these advances, rPPG-based HR detection remains highly sensitive to skin-tone variations, lighting changes, and motion artifacts^4^. Such vulnerabilities make dataset diversity essential for equitable and accurate performance across demographic groups. Our audit of publicly available rPPG datasets indeed reveals significant ethnic and gender biases.

Building on the findings of Lee et al.^5^, extremely high-absorption (dark) or high-reflectance (very light) skin tones can exceed the dynamic-range limits of conventional RGB image sensors, thereby saturating a significant fraction of facial pixels; this saturation diminishes the signal-to-noise ratio of chrominance-based rPPG traces and obscures the subtle photoplethysmographic modulations required for accurate physiological estimation.

Notably, Dasari et al.^4^ evaluated the sensitivity of different rPPG architectures to skin tone and reported that:

- **Traditional chrominance-based methods** incur a mean absolute error (MAE) of 5.2 bpm on Fitzpatrick I-III subjects, which degrades to 14.1 bpm on Fitzpatrick V-VI subjects (*p* < 0.01).
- **Deep-learning models** exhibit a smaller MAE increase, from 6.0 bpm to 9.5 bpm, over the same skin-tone range.

This head-to-head performance comparison demonstrates that, while modern neural approaches partially mitigate skin-tone biases, demographic imbalances in public datasets still translate into clinically relevant accuracy gaps.

**Fig. 1:**
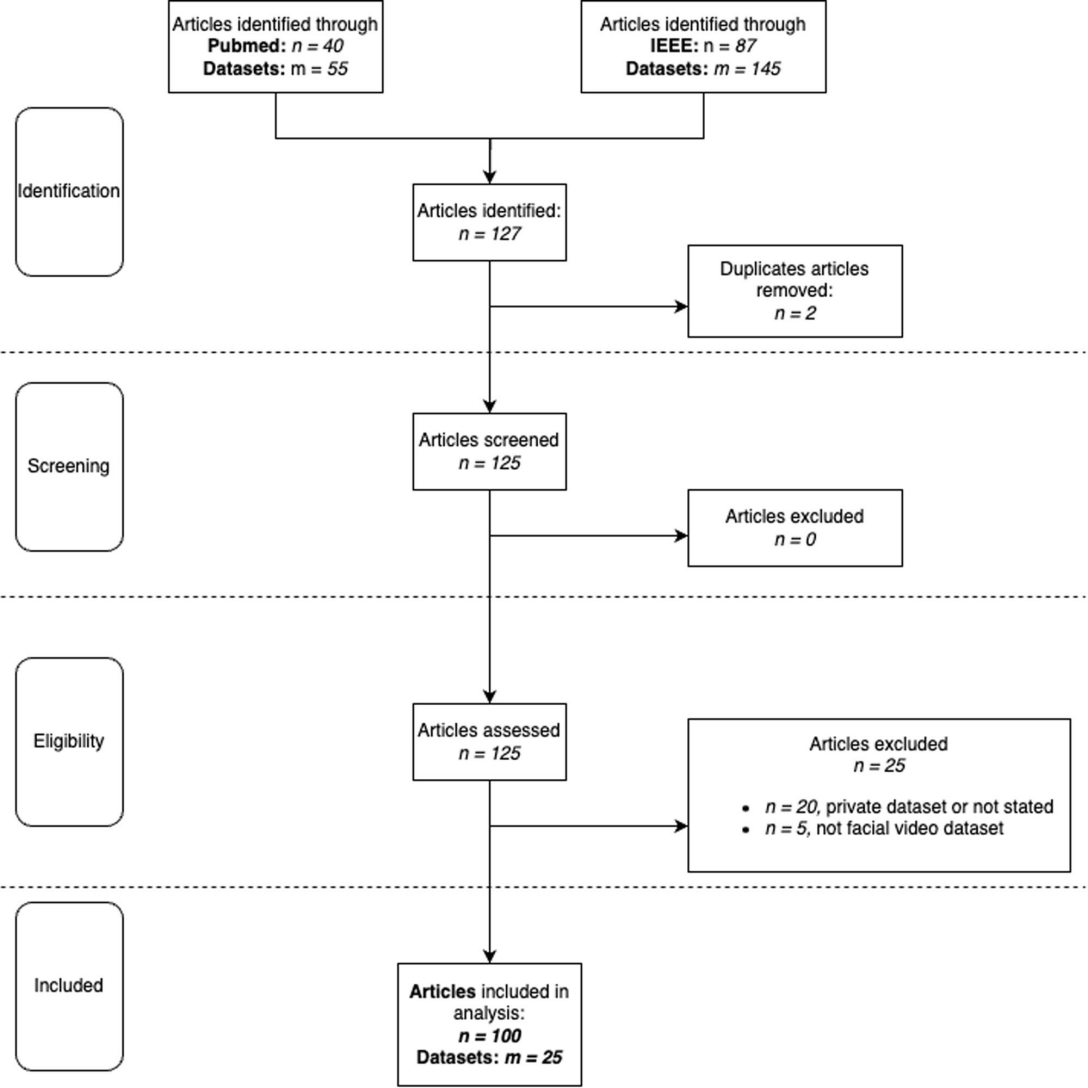
Flow chart of identification, eligibility and inclusion criteria. *m* corresponds to the number of analyzed datasets, *n* corresponds to the number of analyzed articles.

Earlier analyses have individually examined (i) algorithmic advances^6^, (ii) dataset curation^7^, (iii) fairness metrics in physiology^4^, (iv) privacy preserving^8^, or (v) dynamic ROIs selection for various algorithms^9^ with noise assessment techniques^10^. Our work *unifies* these threads by (a) cataloging the demography of *all* public rPPG datasets, and (b) mapping those demographics onto the *performance landscape of competing model families*. This two-axis perspective allows us to ask: *"Which architectures fail, for whom, and under what recording conditions?”*—a question that, to our knowledge, has not been answered systematically in the rPPG literature. To do this, we followed PRISMA^11^ guidelines to conduct a review of 100 studies that utilized public datasets for rPPG-based HR detection (see Fig. 1).

The majority of datasets used in rPPG research predominantly feature individuals with fair skin tones, primarily of European or East Asian descent. These datasets include widely used options, such as UBFC-rPPG, PURE, VIPL-HR, and COHFACE, which are utilized in a significant number of papers. Conversely, datasets comprising multi-ethnic subjects, such as BP4D+, MMSE-HR, and V4V, are underutilized despite their potential to mitigate biases and improve generalization across diverse populations.

This uneven distribution poses challenges in creating ML models that perform equitably across all skin tones. Datasets characterized by underrepresentation of darker skin tones or broad ethnic diversity limit the reliability and fairness of rPPG algorithms in real-world applications.

## Ethnicity representation across rPPG datasets

**Fig. 2:**
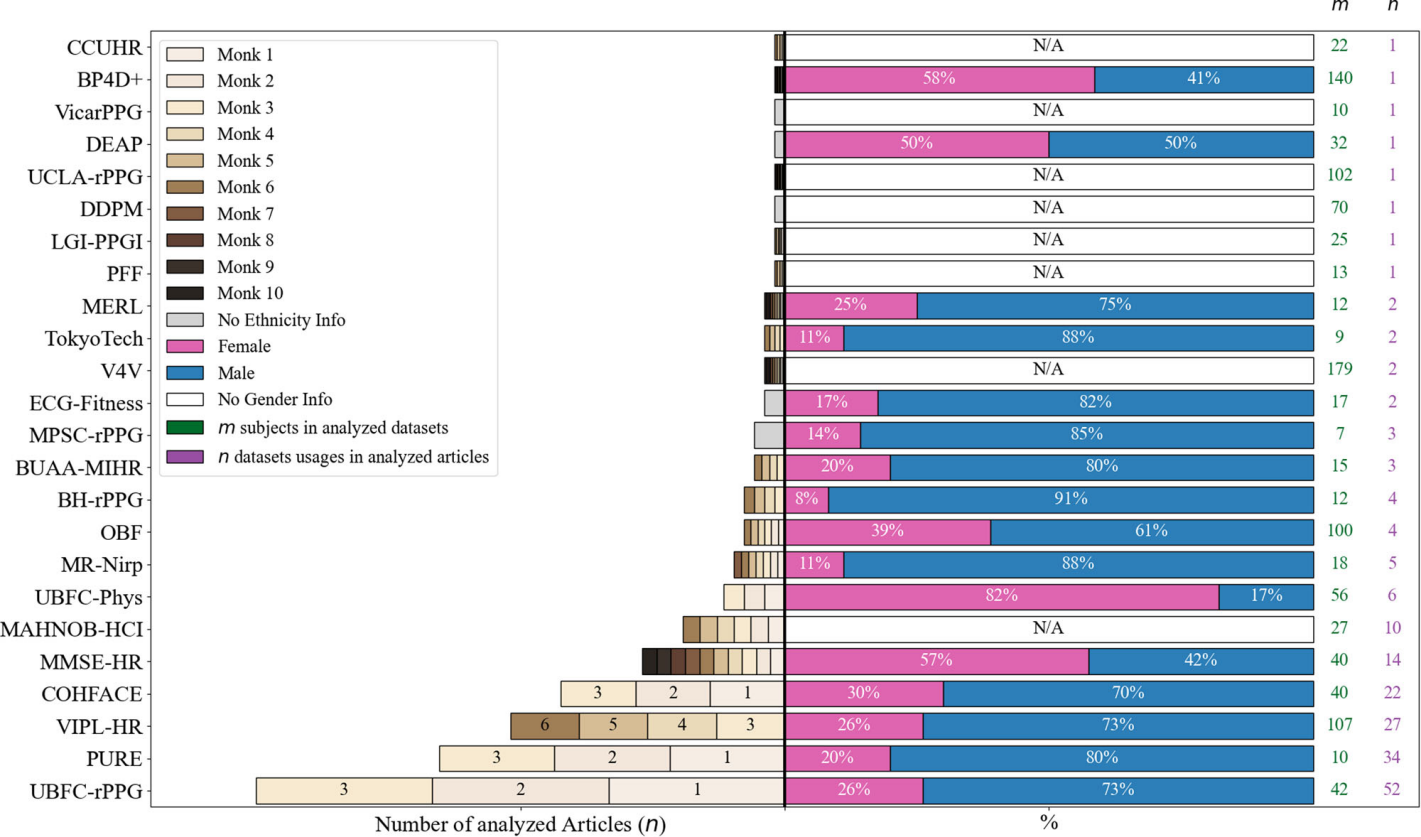
Ethnicity and gender distribution across the datasets examined in the articles. (left part): ethnicity distribution across examined articles visualized in Monk color categories^17^, sorted by the datasets usage in the articles (right part): gender distribution across examined datasets. *m* corresponds to the number of subjects in analyzed datasets, *n* corresponds to the dataset usages in analyzed articles. “N/A" states for datasets, where gender distribution is unknown.

**Fig. 3:**
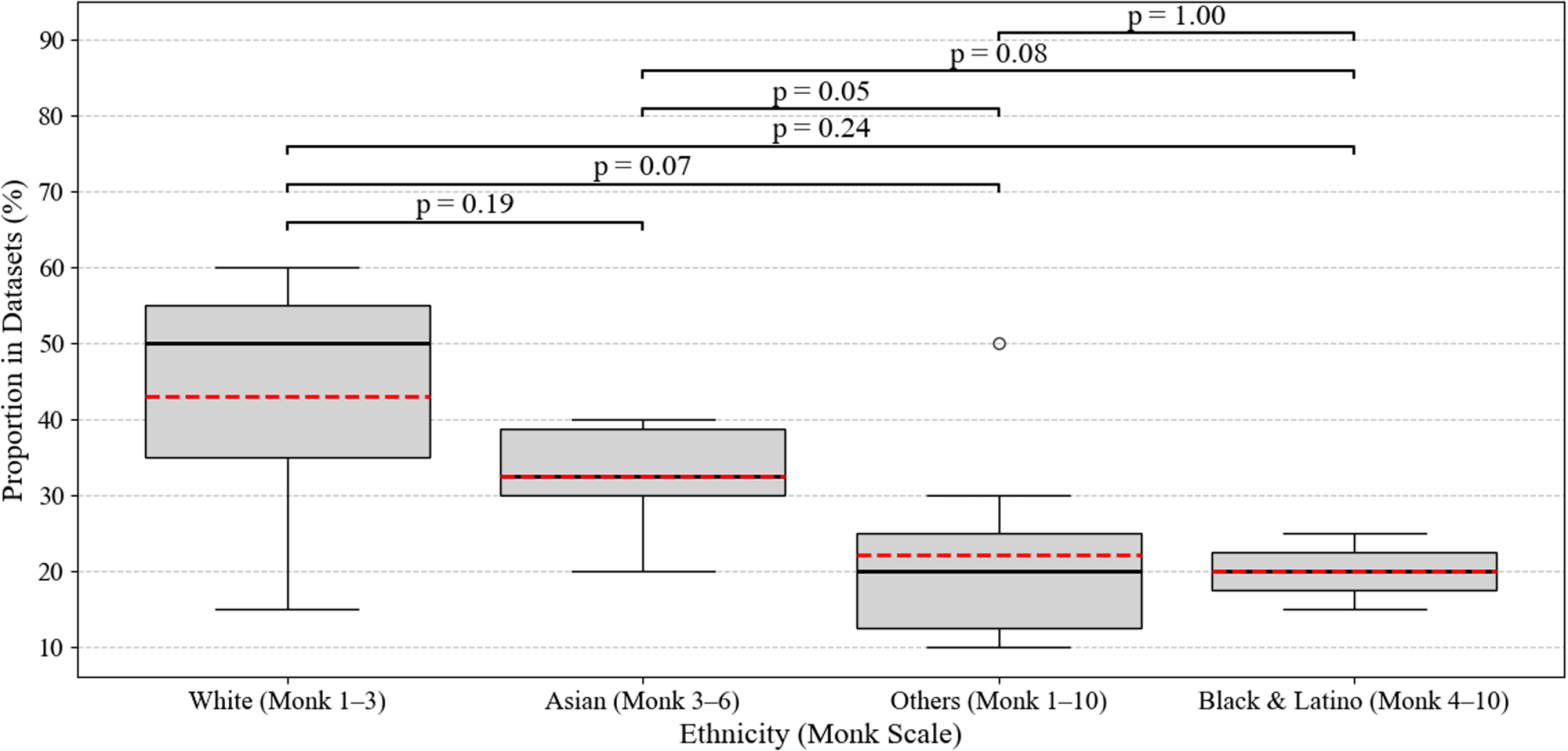
Box plots of ethnic proportion in the analyzed public datasets. Pairwise *p*-values were calculated using the two-sided Mann-Whitney U test between ethnic groups based on the “Proportion in Datasets (%)” distributions.

The majority of publicly available remote photoplethysmography (rPPG) datasets are heavily skewed toward individuals of European descent with lighter skin tones. As summarized in Table 1, datasets such as UBFC-rPPG^12^ (used in 26% of the analyzed studies), PURE^13^ (17%), and COHFACE^14^ (11%) predominantly feature White subjects. VIPL-HR^15^, another frequently used dataset, consists primarily of Asian participants—who also largely fall within the lighter skin tone spectrum. Detailed information on the distribution of skin tone and gender across datasets is presented in Fig. 2, while the ethnic composition of the examined datasets is shown in Fig. 3.

**Table 1:** Overview of the public datasets used in the reviewed rPPG studies

Dataset Name	Year	# Subjects (M/F)	Age (years)	# Videos	Video Length	Video Cameras	FPS	Resolution	HR Measurement Device	Filming Setup	Recording Conditions
UBFC-rPPG^12^	2017	42 (11F, 31M)	*N/A*	42	1 min	Logitech C920 HD Pro	30	640 × 480	CMS50E transmissive pulse oximeter	Indoors, varying sunlight, while solving quiz	1m from camera
PURE^13^	2014	10 (2F, 8M)	*N/A*	60	1 min	eco274CVGE	30	640 × 480	Pulox CMS50E	6 setups: steady, talking, slow/fast translation, slow/medium rotation	Avg. 1.1m, daylight
COHFACE^14^	2016	40 (12F, 28M)	35.6 avg	160	1 min	Logitech HD C525	20	640 × 480	SA9308, SA9311M	*N/A*	Frame-rate 20Hz
MMSE-HR^19^	2016	40 (23F, 17M)	*N/A*	102	30–60 sec	RGB 2D Camera	25	1040 × 1392	*N/A*	Emotional stimuli	*N/A*
VIPL-HR^15^	2018	107 (28F, 79M)	*N/A*	2378	30 sec	Logitech C310, RealSence F200, Huawei P9	25-30	960 × 720 / 1920 × 1080	CMS60C BVP sensor	9 conditions; head movements, varied illumination	1m from camera
LGI-PPG^25^	2018	25 (5F, 20M)	25–42	100	2 min	Logitech HD C270	25	*N/A*	CMS50E PPG	4 conditions; various illumination, motions, talking, bicycle	*N/A*
MAHNOB-HCI^26^	2011	27	*N/A*	527	1-3 min	Allied Vision Stingray F-046C, F-046B	61	780 × 580	*N/A*	Emotional stimulation	Multi-signal setup
MR-Nirp (auto)^27^	2020	18 (2F, 16M)	20–60	190	2 min	FLIR Grasshopper 3 GS3-PGE-23S6C-C	30	640 × 640	CMS50D+	Different weather conditions and motions inside a car	*N/A*
MR-Nirp (indoor)^28^	2018	8 (2F, 6M)	20–40	15	3 min	Point Grey Flea3 FL3-U3-13E4C-C; FLIR Blackfly BFLY-U3-23S6C-C	30	640 × 640	CMS50D+	still and motion experiments	*N/A*
PFF^29^	2017	13	*N/A*	85	3 min	Nikon D5300	50	1280 × 720	MIO Alpha II	5 scenarios	different lighting
OBF^23^	2018	100 (39F, 61M)	18–68	200	5 min	Blackmagic URFA Mini	60	1920 × 1080	NX-EXG2B	Exercise and rest	Symmetric lighting
UBFC-Phys^22^	2021	56 (46F, 10M)	19–38	56	3 min	EO-23121C RGB	35	1024 × 1024	Empatica E4 wristband	Rest, speech, arithmetic tasks	*N/A*
BP4D+^20^	2016	140 (82F, 58M)	18–66	*N/A*	*N/A*	*N/A*	*N/A*	*N/A*	*N/A*	10 emotion tasks	*N/A*
TokyoTech^30^	2019	9 (1F, 8M)	20–60	27	3 min	RGB-NIR Camera	30	640 × 480	Procomp Infinity T7500M	Relax, exercise, relax sessions	*N/A*
CCUHR^31^	2023	22	*N/A*	116	10–20 sec	Intel RealSense D435	30	640 × 480	BIOPAC PPG 100C	Motion vs non-motion scenarios	RGB + NIR Camera
MPSC-rPPG^32^	2022	7 (1F, 6M)	*N/A*	10	5 min	Canon D3500	30	*N/A*	Empatica E4	Sitting idly in lab	Under artificial light
BH-rPPG	2021	12 (1F, 11M)	mean: 32	36	*N/A*	Logitech HD pro webcam C310	20	*N/A*	CMS50E	*N/A*	2 light sources
BUAA-MIHR^33^	2021	15 (3F, 12M)	18–30	165	1 min	Logitech HD pro webcam C930E	30	640 × 480	CMS50E	*N/A*	various illumination
V4V	2021	179	18–66	1300	*N/A*	Di3D (3D Dynamic Imaging System)	25	1040 × 1392	Biopac MP150	*N/A*	symmetric lighting system
DDPM^34^	2021	70	*N/A*	*N/A*	13h totally	*N/A*	*N/A*	*N/A*	*N/A*	RGB, NIR frames and meta-data	*N/A*
UCLA-rPPG^35^	2022	102	Various ages	503	1 min	*N/A*	*N/A*	*N/A*	*N/A*	*N/A*	*N/A*
Vicar-PPG^36^	2014	10	20–35	*N/A*	90 sec	*N/A*	30	720 × 1280	CMS50	*N/A*	*N/A*
ECG-Fitness^37^	2018	17 (3F, 14M)	20–53	204	1 min	Two Logitech C920	30	1920 × 1080	Viatom CheckMeTMPro	4 Fitness activities in 3 lighting setups	Multiple camera angles
MERL^38^	2018	12 (3F, 9M)	20–40	*N/A*	3 min	RGB-Camera: FLIR Blackfly BFLY-U3-23S6C-C; NIR-Camera: GS3-U3-41C6NIR-C	30	640 × 640	CMS 50D+	Controlled lab conditions	Two Bosch EX12LED-3BD-9W illuminators
DEAP^39^	2011	32 (16F, 16M)	19–37	40	1 min	Sony DCR-HC27E	*N/A*	*N/A*	Biosemi ActiveTwo	Controlled lab setup	Watching music videos

Figure 3 further underscores the imbalance in ethnic representation across publicly available rPPG datasets. Individuals with fair skin tones—categorized as White (Monk 1-3)—are markedly overrepresented, with a median dataset proportion nearing 45%. In contrast, participants categorized as Black & Latino (Monk 4-10) exhibit significantly lower representation, with median values below 25%. Pairwise comparisons using the Mann–Whitney *U* test revealed marginally significant differences, particularly between the White and Black & Latino groups (*p* = 0.05). These findings highlight a persistent demographic bias in rPPG datasets, which may undermine model generalizability and performance for individuals with darker skin tones, raising concerns about equity and clinical reliability in real-world deployment.

In our study, we aimed to visualize the usage of various ethnicities by approximating them to the six skin-tone categories of the Fitzpatrick scale^16^ and ten categories Monk Skin Tone Scale^17^. Since no official or universally accepted mapping exists, we adopted the following heuristic rules purely for illustrative purposes, adapted from the work of J. D’Orazio et al.^18^. We stress that this mapping is *not* precise and may overrepresent lighter skin tones (types I-IV), while underrepresenting others, depending on the original dataset labels.

As illustrated in Table 2, whenever a dataset was labeled with keywords such as *"White”* or *"Asian,”* one or more categories (e.g., I-II for White, II-IV for Asian in Fitzpatrick Skin Scheme or 1-3, 3-6 in Monk Skin Scale, accordingly) or were assigned. If multiple keywords appeared (e.g., *"White, Asian”*), we took the union of their respective Monk types, ensuring the maximum possible overlap did not exceed all six types. Lastly, datasets with labels such as *"varying skin tones”* or *"N/A”* were assigned a default set of all ten Monk categories or a neutral color for “no ethnicity data.”

**Table 2:** Mapping of Monk Skin Tone Scale^17^ to Fitzpatrick Skin Type^16^ with Descriptions and Associated Ethnicities/Regions adapted from D’Orazio et al.^18^

Monk Skin Tone Scale	Fitzpatrick Skin Type	Skin Tone Description	Associated Ethnicities/Regions
1	I	Very fair skin, always burns, never tans	Northern European (e.g., Celtic)
2	I	Fair skin, burns easily, tans minimally	Northern European
3	II	Light skin, burns moderately, tans gradually	European, Asian
4	III	Medium skin, may experience mild burns, tans uniformly	Southern European, Middle Eastern, Hispanic
5	III	Olive skin, rarely burns, tans easily	Mediterranean, Middle Eastern, Asian
6	IV	Brown skin, rarely burns, tans darkly easily	Hispanic, Middle Eastern, Asian, Indigenous peoples
7	IV	Dark brown skin, very rarely burns, tans very easily	African, African-American, Pacific Islander
8	V	Deeply pigmented dark brown skin, never burns	African, African-American, Aboriginal Australian
9	V	Very dark brown skin, never burns	African, African-American
10	VI	Darkest brown to black skin, never burns	African, African-American

Ethnicity labels (e.g., “Asian”) conflate geography, culture, and phenotype; they do not uniquely determine melanin content. Our heuristic, adapted from D’Orazio et al.^18^, therefore introduces classification noise, especially for mixed-heritage participants. However, future data sets should report direct skin tone measures (e.g., handheld colorimeter values or Monk self-assessment cards) to remove this source of uncertainty. Rater A (M.B.) and Rater B (M.E.) independently evaluated the skin tone of each subject using the 10-point Monk Skin Tone Scale in random frames of subjects from the PURE^13^ dataset. The agreement between the raters was high, with 100% of the ratings falling within a ± 1 difference. The mean absolute error was 0.44, corresponding to an average deviation of 4.4%. Although one rater consistently used a single score, the minimal differences observed indicate strong practical alignment between independent assessments. Although the sample is small, the result suggests that our light/medium/dark grouping is not dominated by subjective error. However, for future studies, such heuristic validation should be performed with various datasets of different subjects on a larger scale.

Only a few datasets, such as MMSE-HR^19^ and BP4D+^20^, provide some degree of ethnic diversity. MMSE-HR explicitly includes subjects categorized within the Fitzpatrick skin type scale (II-VI), exhibiting representation of darker skin tones, albeit with only a small portion of subjects classified as types V and VI. The BP4D+ dataset, which included individuals from Hispanic, Black, and Asian backgrounds, was one of the few datasets promoting ethnic diversity. However, its use remained limited, appearing in fewer than five percent of the analyzed studies.

This lack of diversity is problematic because skin tone influences the reflectance of light captured in rPPG signals. Due to higher melanin concentrations in darker skin tones, they reflect less light, which can reduce signal intensity and lead to higher HR estimation errors. ML models trained predominantly on lighter-skinned individuals may fail to generalize well for darker-skinned individuals, leading to biased HR readings.

Nowara et al.^7^, in their meta-analysis, reported a substantial performance degradation in remote photoplethysmography (rPPG) accuracy for individuals with darker skin tones. Specifically, they observed an increase in mean absolute error (MAE) from 4.23 bpm for Fitzpatrick skin types I-V to 13.58 bpm for type VI, reflecting a more than twofold degradation in performance. Additionally, they noted a slight reduction in accuracy for females, with an MAE of 4.49 compared to 3.78 for males on the CHROME dataset. Similarly, Comas et al.^21^ confirmed this degradation trend and highlighted the potential of data augmentation techniques to mitigate such disparities.

## Gender representation and its effects on model fairness

Another notable bias in rPPG datasets relates to gender distribution. While many datasets aim for a balanced representation, our findings indicate that some datasets contained significantly more female subjects than male, as illustrated in Fig. 2 (right part). For instance, UBFC-Phys^22^ (46 females, 10 males) and BP4D+ (82 females, 58 males) presented a reversed imbalance that favored female subjects. In contrast, datasets such as VIPL-HR^15^ and OBF^23^ were characterized by a strong male dominance.

Gender imbalance can introduce algorithmic biases in HR estimation, particularly because physiological differences (such as skin vascularization and hormone-driven fluctuations) may impact rPPG signal characteristics. Therefore, models trained on gender-imbalanced datasets may exhibit varying accuracy levels across populations.

Following Charkoudian et al.^24^, resting internal temperature increases in women in the midluteal phase of the menstrual cycle, when progesterone and estrogen are elevated, compared with the early follicular phase when these hormones are low. Conversely, men tend to have thicker facial epidermis and a higher prevalence of facial hair, both of which attenuate or occlude the green-channel pulsatile signal. These optical and hemodynamic contrasts motivate a separate error analysis by gender.

## Recommendations for fair and inclusive rPPG research

1. **Increase Dataset Diversity**: Researchers should prioritize the inclusion of ethnically diverse participants in public datasets. Efforts should be made to balance skin tone representation across the Monk Skin Tone Scale.
2. **Standardized Reporting of Ethnicity and Skin Tone**: Many studies fail to report skin tone or ethnicity information, making it difficult to evaluate dataset diversity. Future research should adopt standardized reporting practices for demographic characteristics to improve transparency and reproducibility.
3. **Balanced Gender Representation**: Studies should ensure a balanced distribution of male and female participants to mitigate gender-related biases in HR detection algorithms.
4. **Adapting ML Models for Diverse Populations**: Researchers should explore adaptive learning techniques, such as domain adaptation and fairness-aware ML algorithms, to improve model robustness across demographic groups.
5. **Benchmarking on Inclusive Datasets**: New rPPG HR detection algorithms should be tested on datasets that represent diverse ethnic and gender populations to ensure unbiased performance evaluations.
6. **Dataset Scale and Protocol Harmonization**: Most publicly available rPPG datasets remain one to two orders of magnitude smaller than the vision datasets typically used to train deep models. Limited subject count and recording diversity make it difficult to capture the full variance in skin tone, age, and motion, hampering generalization and fairness. We therefore see an urgent need for (i) larger, multi-site datasets (>1k participants) collected under harmonized protocols, (ii) diversity of ethnical and gender background with precise documentation (e.g., using Monk Skin Tone Scale), and (iii) synthetic data or self-supervised pre-training to bridge the sample-efficiency gap.
7. **Ethnic Biases of Traditional vs ML-based methods**: Undertake a systematic investigation of the extent to which demographic factors influence the performance of distinct rPPG model architectures, including both traditional signal-processing methods and modern deep-learning approaches.

The lack of diversity in public rPPG datasets presents a significant challenge to the fairness and accuracy of ML-based heart rate detection models. Our review reveals a strong bias toward subjects with fair skin tones in most data sets, accompanied by a limited representation of individuals with darker skin tones and noticeable gender imbalances. These biases can lead to reduced performance and fairness concerns, particularly in clinical and healthcare applications. Addressing these issues requires a concerted effort from researchers to improve dataset inclusivity, standardize demographic reporting, and develop ML models that account for diversity. Ensuring equitable representation in rPPG datasets is essential to advance reliable and fair HR monitoring technologies for all populations.

## Methods

### Study design and article selection

We conducted a systematic review of 100 peer-reviewed studies that employed publicly available datasets for rPPG-based heart rate (HR) detection. The selection process followed PRISMA guidelines^11^, and included articles sourced from PubMed and IEEE Xplore databases using a defined combination of search terms. Inclusion criteria required studies to use facial video for rPPG and to report dataset usage details.

### Dataset categorization and metadata extraction

For each dataset used in the selected studies, we extracted metadata including the number of subjects, gender distribution, age range (if available), type of RGB camera used, frame rate, resolution, HR ground truth device, and recording conditions. These attributes were tabulated and are summarized in Table 1.

### Skin tone mapping using Fitzpatrick and Monk scales

To analyze ethnicity distribution, we used heuristic mappings of ethnic labels (e.g., “White”, “Asian”) to both Fitzpatrick skin types (I–VI) and Monk Skin Tone categories (1–10). The approach was adapted from D’Orazio et al.^18^, allowing us to approximate demographic representation even when direct skin tone data was unavailable. Table 2 describes the mapping scheme.

### Skin tone validation and rater agreement

Two raters (M.B. and M.E.) independently assessed skin tone on a random sample of subjects from the PURE dataset^13^, using the 10-point Monk Skin Tone Scale. Ratings were compared by computing the mean absolute error and by calculating the proportion of ratings that fell within ± 1 unit. This validation step helped ensure that heuristic-based ethnicity assignments did not introduce substantial subjective error.

### Statistical analysis

To quantify demographic imbalance, we computed the distribution of Monk Skin Tone categories and gender across datasets. Differences between ethnic groups were tested using the Mann-Whitney U test. Visualizations include flow charts (Fig. 1), bar graphs (Fig. 2), and box plots (Fig. 3).

## Supplementary information

Supplementary information

## Data Availability

All data supporting the findings of this study are provided within the paper and on Zenodo (10.5281/zenodo.15075947). The code supporting the findings of this study is available within the paper and through the GitHub repository (https://github.com/Maksym-Bondarenko/rppg-ethnicity-paper).
