# Supplementary material for: Demographic bias in public remote photoplethysmography datasets
Source: NPJ Digit Med. 2025 Oct 2;8:593. doi: 10.1038/s41746-025-01973-9 (PMC12491395; doi:10.1038/s41746-025-01973-9)
Supplement: Supplementary file 1 — Supplementary information [file 41746_2025_1973_MOESM1_ESM.pdf]

# Supplementary Information

**Table S1.** Overview of the 100 rPPG papers examined in this survey.

| Paper title | Year | Authors | Public datasets used |
|---|---|---|---|
| A Review of Deep Learning-Based Contactless Heart Rate Measurement Methods | 2021 | Aoxin Ni et al. | UBFC-RPPG |
| Real-time realizable mobile imaging photoplethysmography | 2022 | Hooseok Lee et al. | UBFC-rPPG |
| Performance analysis of remote photoplethysmography deep filtering using long short-term memory neural network | 2022 | Deivid Botina-Monsalve et al. | MMSE-HR, VIPL-HR, COHFACE |
| Heart rate prediction from facial video with masks using eye location and corrected by convolutional neural networks | 2022 | Kun Zheng et al. | PFF, PURE, UBFC-rPPG + masks |
| Deep learning-based remote-photoplethysmography measurement from short-time facial video | 2022 | Bin Li et al. | OBF, COHFACE, UBFC-rPPG |
| LSTM-based real-time signal quality assessment for blood volume pulse analysis | 2023 | Haoyuan Gao et al. | UBFC-Phys, LGI-PPGI |
| Contactless Cardiovascular Assessment by Imaging Photoplethysmography: A Comparison with Wearable Monitoring | 2023 | Valerie A A van Es et al. | UBFC-rPPG |
| Evaluating Visual Photoplethysmography Method | 2022 | Debjyoti Talukdar et al. | UBFC-Phys |
| Heart Rate Estimation from Facial Image Sequences of a Dual-Modality RGB-NIR Camera | 2023 | Wen-Nung Lie et al. | PURE, CCUHR |
| Remote photoplethysmography (rPPG) in the wild: Remote heart rate imaging via online webcams | 2024 | Daniele Di Lernia et al. | COHFACE |
| Shielding facial physiological information in video | 2022 | Kun Zheng et al. | VIPL-HR |
| Robust Heart Rate Variability Measurement from Facial Videos | 2023 | Ismoil Odinaev et al. | UBFC-rPPG |
| Heart rate estimation network from facial videos using spatiotemporal feature image | 2022 | Kokila Bharti Jaiswal et al. | MAHNOB-HCI, MMSE-HR, UBFC-rPPG, VIPL-HR |
| Remote Heart Rate Prediction in Virtual Reality Head-Mounted Displays Using Machine Learning Techniques | 2022 | Tiago Palma Pagano et al. | MR-Nirp (auto + indoor), UBFC-rPPG |
| Remote photoplethysmography with constrained ICA using periodicity and chrominance constraints | 2018 | Richard Macwan et al. | UBFC-rPPG, MMSE-HR |
| Hierarchical Style-Aware Domain Generalization for Remote Physiological Measurement | 2024 | Jiyao Wang et al. | VIPL-HR, V4V, PURE, BUAA, UBFC-rPPG |
| Weighted combination and singular spectrum analysis based remote photoplethysmography pulse extraction in low-light environments | 2022 | Lin Xi et al. | PURE, MIHR |
| Heart Rate Measurement Based on 3D Central Difference Convolution with Attention Mechanism | 2022 | Xinhua Liu et al. | PURE, UBFC-rPPG |
| Robust Remote Photoplethysmography Estimation With Environmental Noise Disentanglement | 2024 | Si-Qi Liu et al. | PURE, COHFACE, UBFC-rPPG, MMSE-HR, MAHNOB-HCI |
| Facial Video-Based Non-Contact Stress Recognition Utilizing Multi-Task Learning With Peak Attention | 2024 | Juncong Xu et al. | UBFC-Phys |

*Continued on next page*

*Table S1 continued from previous page*

| **Paper title** | **Year** | **Authors** | **Public datasets** |
|---|---|---|---|
| Remote photoplethysmography based on reflected light angle estimation | 2024 | Xuanhe Fan et al. | PURE |
| Innovative approaches in imaging photoplethysmography for remote blood oxygen monitoring | 2024 | Shangwei Zhu et al. | VIPL-HR |
| Heart rate estimation from facial videos with motion interference using T-SNE-based signal separation | 2022 | Hequn Wang et al. | UBFC-rPPG, VIPL-HR |
| An Evaluation of Non-Contact Photoplethysmography-Based Methods for Remote Respiratory Rate Estimation | 2023 | Giuseppe Boccignone et al. | BP4D+ |
| An effective cross-scenario remote heart rate estimation network based on global-local information and video transformer | 2024 | Guoliang Xiang et al. | UBFC-rPPG, PURE |
| 3D DenseNet with temporal transition layer for heart rate estimation from real-life RGB videos | 2025 | Mohammad Khaleel Sallam Ma'aitah et al. | VIPL-HR |
| Learning Spatio-Temporal Pulse Representation With Global-Local Interaction and Supervision for Remote Prediction of Heart Rate | 2024 | Changchen Zhao et al. | UBFC-rPPG, PURE |
| A Comparative Survey of Methods for Remote Heart Rate Detection From Frontal Face Videos | 2018 | Chen Wang et al. | MAHNOB-HCI |
| Assessment of Deep Learning-Based Heart Rate Estimation Using Remote Photoplethysmography Under Different Illuminations | 2022 | Ze Yang et al. | UBFC-rPPG, BH-rPPG |
| Application of Chrominance Based rPPG in Estimation of Heart Rate from Video Signal | 2021 | A.H.M. Zadidul Karim et al. | UBFC-rPPG |
| Remote photoplethysmography signals enhancement based on generative adversarial networks | 2023 | Hailan Kuang et al. | UBFC-rPPG, PURE |
| Efficient Real-Time Camera Based Estimation of Heart Rate and Its Variability | 2019 | Amogh Gudi et al. | VicarPPG, PURE |
| Heart Rate Estimation From Remote Photoplethysmography Based on Light-Weight U-Net and Attention Modules | 2023 | Sung-Nien Yu et al. | UBFC-rPPG |
| Remote Photoplethysmography Using Nonlinear Mode Decomposition | 2018 | Halil Demirezen et al. | PURE |
| ReViSe: Remote Vital Signs Measurement Using Smartphone Camera | 2022 | Donghao Qiao et al. | TokyoTech rPPG, PURE |
| Influence of ROI Selection for Remote Photoplethysmography with Singular Spectrum Analysis | 2021 | GuoPing Wang et al. | COHFACE |
| Comparison of Region of Interest Segmentation Methods for Video-Based Heart Rate Measurements | 2018 | Peixi Li et al. | UBFC-rPPG |
| Improved Heart Rate Estimation From Facial Videos Using Hair Detection and Majority Vote in Subintervals | 2023 | Panupong Sunkom et al. | UBFC-rPPG |
| Self-rPPG: Learning the Optical & Physiological Mechanics of Remote Photoplethysmography with Self-Supervision | 2022 | Zahid Hasan et al. | MPSC-rPPG, MERL, UBFC-rPPG |
| SrPPG: Semi-Supervised Adversarial Learning for Remote Photoplethysmography with Noisy Data | 2023 | Zahid Hasan et al. | MPSC-rPPG, MERL, UBFC-rPPG |

*Continued on next page*

*Table S1 continued from previous page*

| **Paper title** | **Year** | **Authors** | **Public datasets** |
|---|---|---|---|
| Video Segment Time-Channel Frequency Attention Network for Remote Photoplethysmography | 2024 | Min Hu et al. | PURE, COHFACE, MAHNOB-HCI |
| Uncertainty Quantification for Deep Learning-Based Remote Photoplethysmography | 2023 | Rencheng Song et al. | UBFC-rPPG, PURE, MAHNOB-HCI, VIPL-HR |
| Remote Heart Rate Estimation in Intense Interference Scenarios: A White-Box Framework | 2024 | Boxiang Liu et al. | <UBFC-rPPG, PURE |
| Deep Super-Resolution Network for rPPG Information Recovery and Noncontact Heart Rate Estimation | 2021 | Zijie Yue et al. | DEAP |
| Facial Video-based Remote Photoplethysmography Signal Estimation with Vision Transformer | 2023 | Chae-Min Kim et al. | MPSC-rPPG |
| ETA-rPPGNet: Effective Time-Domain Attention Network for Remote Heart Rate Measurement | 2021 | Min Hu et al. | PURE, COHFACE, UBFC-rPPG, MMSE-HR |
| A Multiresolution Method for Non-Contact Heart Rate Estimation Using Facial Video Frames | 2022 | Mousumi Das et al. | COHFACE |
| Development and Evaluation of a Contactless Heart Rate Measurement Device Based on rPPG | 2022 | Nur Ahmadi et al. | VIPL-HR |
| Deep-Learning-Based Remote Photoplethysmography Measurement in Driving Scenarios With Color and Near-Infrared Images | 2023 | Li-Wen Chiu et al. | MR-NIRP (car) |
| A feasibility study of a video-based heart rate estimation method with convolutional neural networks | 2019 | Senle Zhang et al. | MAHNOB-HCI, VIPL-HR, UBFC-rPPG |
| Unrolled iPPG: Video Heart Rate Estimation via Unrolling Proximal Gradient Descent | 2023 | Vineet R. Shenoy et al. | MMSE-HR |
| rPPG-MAE: Self-Supervised Pretraining With Masked Autoencoders for Remote Physiological Measurements | 2024 | Xin Liu et al. | VIPL-HR, PURE, UBFC-rPPG |
| Time–Frequency Learning Framework for rPPG Signal Estimation Using Scalogram-Based Feature Map of Facial Video Data | 2023 | Mousumi Das et al. | UBFC-rPPG, COHFACE, ECG Fitness |
| A Novel Video-Based Real-Time Non-Contact Heart Rate Measurement Algorithm: Based on Third-Order Adaptive FastICA | 2024 | Pengji Chen et al. | UBFC-rPPG |
| Robust Heart Rate Measurement by Adaptive ROI Selection for Head-Rotation Conditions | 2023 | Xuanhe Fan et al. | MR-NIRP (car), PURE |
| LSDASCFormer: A Transformer-Like Network With Long–Short-Distance Attention and SC-Conv for Heart Rate Measurement | 2024 | Xinhua Liu et al. | UBFC-rPPG, PURE |
| Self-Supervised RGB-NIR Fusion Video Vision Transformer Framework for rPPG Estimation | 2022 | Soyeon Park et al. | VIPL-HR, MR-NIR-Car |
| CMRPPGFormer: 3-D Spatio-Temporal Convolutional Modulation Transformer Network for Remote Heart Rate Estimation | 2024 | Xiaolin Ma et al. | UBFC-rPPG, PURE |
| EST-TSANet: Video-Based Remote Heart Rate Measurement Using Temporal Shift Attention Network and ESTmap | 2023 | Rui Zhong et al. | UBFC-rPPG, VIPL-HR |
| AI-Driven rPPG Heart Rate Detection for In-Vehicle Monitoring | 2024 | Martina Pierri et al. | UBFC-rPPG |

*Continued on next page*

*Table S1 continued from previous page*

| Paper title | Year | Authors | Public datasets |
|---|---|---|---|
| Data-Augmentation for Deep Learning Based Remote Photoplethysmography Methods | 2021 | Simon Perche et al. | UBFC-rPPG, COHFACE, VIPL-HR |
| rPPG-Based Heart Rate Estimation Using Spatial-Temporal Attention Network | 2021 | Min Hu et al. | PURE, MMSE-HR, UBFC-rPPG |
| Measuring Heart Rate and Heart Rate Variability with Smartphone Camera | 2021 | Donghao Qiao et al. | TokyoTech |
| Vision-Based Heart Rate Estimation Via A Two-Stream CNN | 2019 | Zhi-Kuan Wang et al. | COHFACE |
| AutoHR: A Strong End-to-End Baseline for Remote Heart Rate Measurement With Neural Searching | 2020 | Zitong Yu et al. | VIPL-HR, MAHNOB-HCI, MMSE-HR |
| Channel-Wise Interactive Learning for Remote Heart Rate Estimation From Facial Video | 2023 | Qi Li et al. | UBFC-rPPG, PURE, MMSE-HR |
| Research on Robust Measurement Method of Heart Rate Using Remote Photoplethysmography Based on Adversarial Learning Network with High and Low Frequency Features | 2025 | Dezhao Zhai et al. | COHFACE, UBFC-rPPG, BUAA-MIHR |
| DelNet based Systolic Peak Delineation in Remote PPG Signal from Facial Video Frames | 2023 | Mousumi Das et al. | UBFC-rPPG |
| Remote Heart Rate Detection Based on 3D-DenseNet and Attention Mechanism | 2024 | Zengfa Wang et al. | COHFACE |
| Non-Contact Heart Rate Measurement From Facial Video Data Using a 2D-VMD Scheme | 2022 | Mousumi Das et al. | COHFACE |
| Robust Remote Heart Rate Estimation from Face Utilizing Spatial-temporal Attention | 2019 | Xuesong Niu et al. | VIPL-HR, MMSE-HR |
| BVPNet: Video-to-BVP Signal Prediction for Remote Heart Rate Estimation | 2021 | Abhijit Das et al. | MMSE-HR, VIPL-HR |
| Deep adaptative spectral zoom for improved remote heart rate estimation | 2024 | Joaquim Comas et al. | UCLA-rPPG, PURE, UBFC-rPPG |
| Heart Rate Estimation from Facial Videos Using 2D Convolution Neural Network | 2024 | Omar Mohamed et al. | PURE |
| CoSTHR: A Heart Rate Estimating Network With Adaptive Color Space Transformation | 2022 | Zhaolin Qiu et al. | VIPL-HR |
| Anti-Motion Remote Measurement of Heart Rate Based on Region Proposal Generation and Multi-Scale ROI Fusion | 2022 | Changchen Zhao et al. | PURE, UBFC-rPPG |
| Deep learning-based image enhancement for robust remote photoplethysmography in various illumination scenarios | 2023 | Shutao Chen et al. | BH-rPPG |
| Illumination Variation-Resistant Network for Heart Rate Measurement by Exploring RGB and MSR Spaces | 2024 | Lili Liu et al. | COHFACE, BH-rPPG, VIPL-HR |
| Competitive State Anxiety Inventory Assessment Using Remote Photoplethysmography and Deep Learning | 2023 | Lin He et al. | UBFC-Phys |
| Video-Based Physiological Measurement Using 3D Central Difference Convolution Attention Network | 2021 | Yu Zhao et al. | COHFACE, PURE, UBFC-rPPG |
| Generalizable Remote Physiological Measurement via Semantic-Sheltered Alignment and Plausible Style Randomization | 2024 | Jiyao Wang et al. | UBFC-rPPG, BUAA, PURE, VIPL-HR, V4V |

*Continued on next page*

*Table S1 continued from previous page*

| Paper title | Year | Authors | Public datasets |
|---|---|---|---|
| Enhanced DeepPhys: Leveraging Deep Learning for Heart Rate Detection from Facial Videos | 2024 | Ebtesam S. Abdelwahab et al. | PURE, UBFC-Phys |
| cbPPGGAN: A Generic Enhancement Framework for Unpaired Pulse Waveforms in Camera-Based Photoplethysmography | 2023 | Ze Yang et al. | BH-rPPG, UBFC-rPPG |
| Temporal Hierarchical Quick Spatial Attention Mechanism for Heart Rate Measurement | 2024 | Minghao Ji et al. | PURE, MAHNOB-HCI |
| Self-Supervised Augmented Vision Transformers for Remote Physiological Measurement | 2023 | Liyu Pang et al. | VIPL-HR |
| ALPINE: Improving Remote Heart Rate Estimation using Contrastive Learning | 2023 | Lokendra Birla et al. | UBFC-rPPG, COHFACE |
| Information-Enhanced Network for Noncontact Heart Rate Estimation From Facial Videos | 2023 | Lili Liu et al. | OBF, DDPM, MR-NIRP |
| MaskFusionNet: A Dual-Stream Fusion Model With Masked Pre-Training Mechanism for rPPG Measurement | 2024 | Yizhu Zhang et al. | VIPL-HR, COHFACE, PURE |
| TranPulse: Remote Photoplethysmography Estimation With Time-Varying Supervision to Disentangle Multiphysiologically Interference | 2024 | Hang Shao et al. | UBFC-rPPG, COHFACE, VIPL-HR, PURE |
| Non-Contact HR Extraction from Different Color Spaces Using RGB Camera | 2022 | Arpita Panigrahi et al. | UBFC-rPPG, COHFACE, PURE |
| Predicting and Classifying Heart Rates Using Instantaneous Video Data | 2023 | Paramita Basak Upama et al. | UBFC-Phys |
| PhysFormer: Facial Video-based Physiological Measurement with Temporal Difference Transformer | 2022 | Zitong Yu et al. | VIPL-HR, MAHNOB-HCI, MMSE-HR, OBF |
| A Comparative Study of Principled rPPG-Based Pulse Rate Tracking Algorithms for Fitness Activities | 2024 | Qiang Zhu et al. | ECG Fitness |
| Style Transfer with Bio-realistic Appearance Manipulation for Skin-tone Inclusive rPPG | 2022 | Yunhao Ba et al. | UBFC-rPPG |
| Periodic Variance Maximization Using Generalized Eigenvalue Decomposition Applied to Remote Photoplethysmography Estimation | 2018 | Richard Macwan et al. | UBFC-rPPG, MMSE-HR |
| An End-to-end Efficient Framework for Remote Physiological Signal Sensing | 2021 | Chengyang Hu et al. | VIPL-HR, OBF |
| A Two-Stream Deep-Learning Network for Heart Rate Estimation From Facial Image Sequence | 2024 | Wen-Nung Lie, et al. | MAHNOB-HCI, MMSE-HR, PURE, UBFC-rPPG |
| RADIANT: Better rPPG estimation using signal embeddings and Transformer | 2023 | Anup Kumar Gupta et al. | UBFC-rPPG, COHFACE |
| TCNTransNet: A Semi-Supervised Temporal-Spatial Fusion Framework for Heart Rate Estimation From Camera Video | 2024 | Bailin Hou et al. | COHFACE, VIPL-HR |
| Noncontact Multiphysiological Signals Estimation via Visible and Infrared Facial Features Fusion | 2022 | Shuai Ding et al. | VIPL-HR, UBFC-rPPG |

2

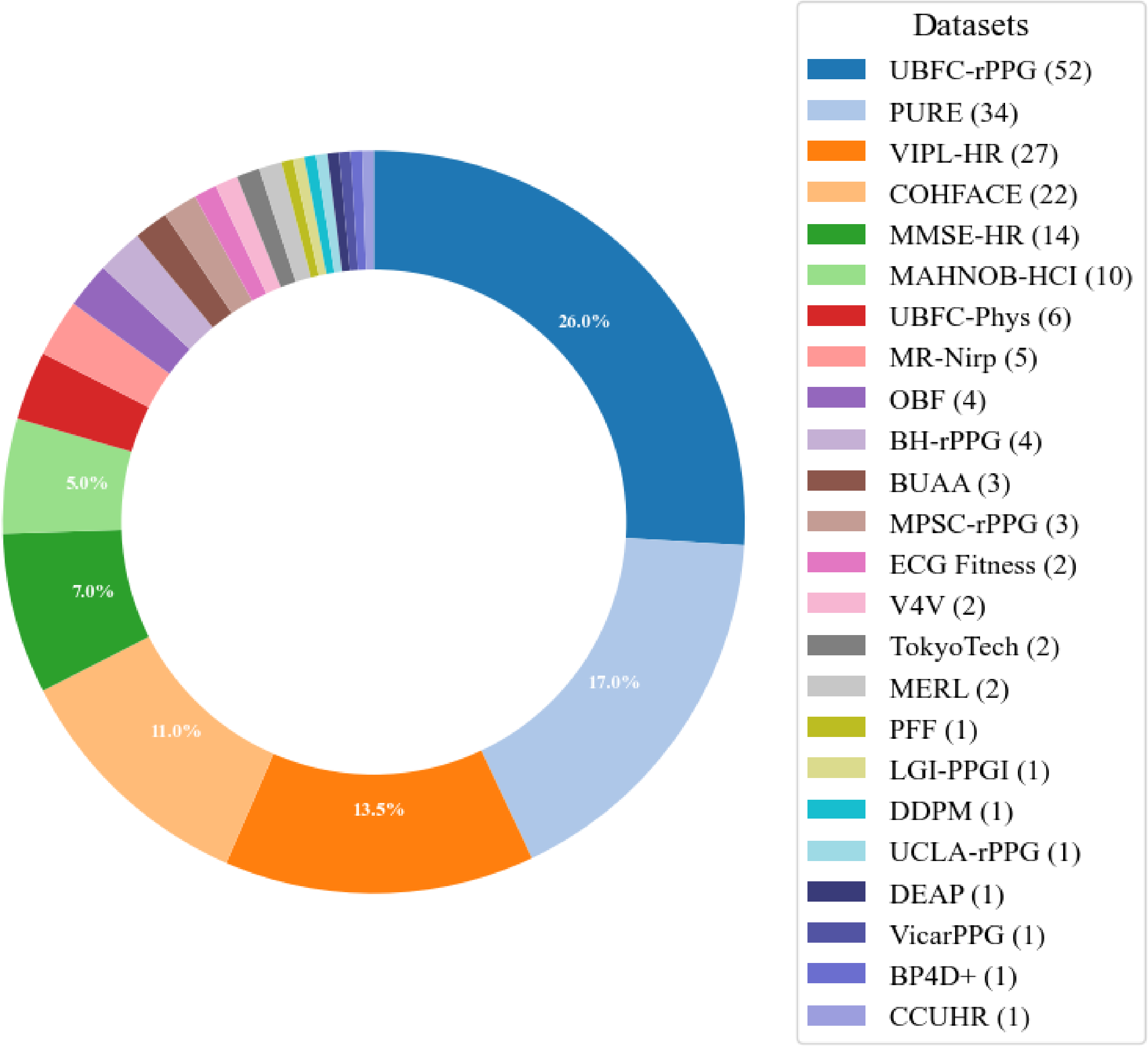


**Figure S1.** Distribution of public datasets across 100 examined articles (from Table S1).

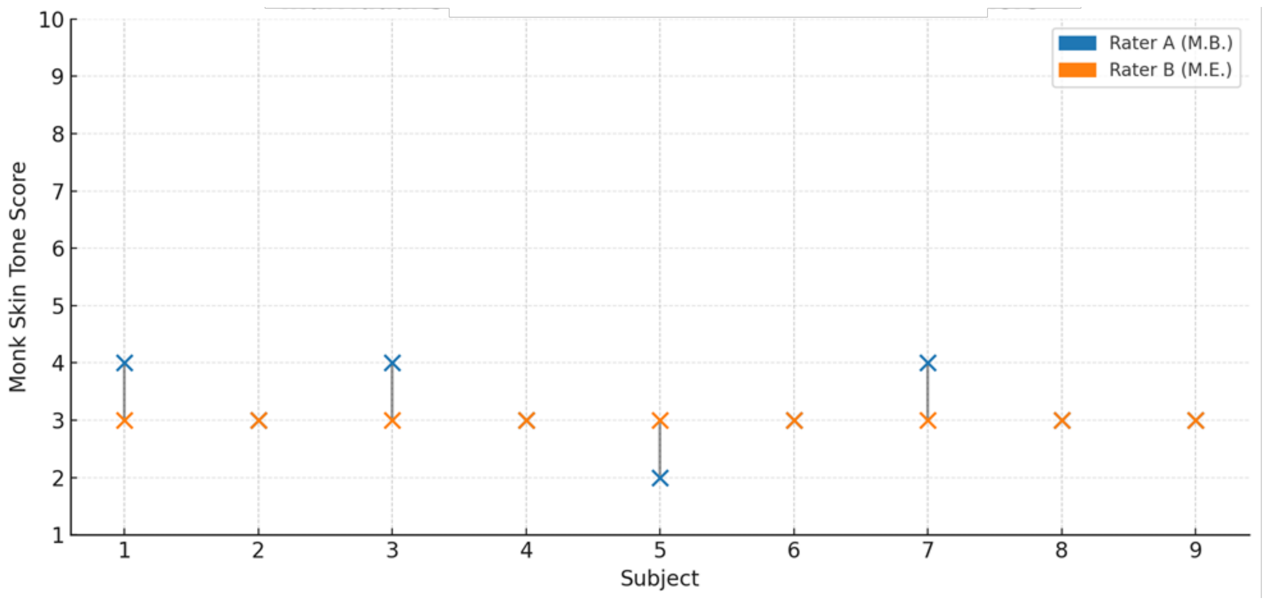


**Figure S2.** Rater A (M.B.) and Rater B (M.E.) independently evaluated the skin tone of each subject using the 10-point Monk Skin Tone Scale. Agreement between raters was high, with 100% of the ratings falling within a ±1 difference. The mean absolute error was 0.44, corresponding to an average deviation of 4.4%. Although one rater consistently used a single score, the minimal differences observed indicate strong practical alignment between independent assessments.
